# Bridging the Lab-to-Clinic Gap in Intranasal Nanomaterial-Based Chemotherapy for Glioblastoma

**DOI:** 10.3390/pharmaceutics18091073

**Published:** 2026-08-27

**Authors:** Sophia Leslie, Stella Rios, Hana Elnahas, Megan Keniry

**Affiliations:** School of Integrative Biological and Chemical Sciences (SIBCS), University of Texas Rio Grande Valley, 1201 W. University Dr., Edinburg, TX 78539, USA

**Keywords:** intranasal delivery, glioblastoma, nose-to-brain, nanomaterials, translational research, drug delivery

## Abstract

Clinical outcomes for brain cancer are often poor because the blood–brain/tumor barrier hinders effective drug delivery to malignant tissue. Glioblastoma, the most common primary brain malignancy in adults, has an average survival of approximately fourteen months. Here, we discuss novel strategies that our research group and others are developing to deliver chemotherapy to the brain via the nasal cavity. Although significant hurdles remain, intranasal delivery holds substantial promise for improving outcomes for patients with brain cancer. Intranasal delivery is noninvasive, permits repeated dosing, and has been shown to enable direct nose-to-brain transport that bypasses the blood–brain barrier. Challenges such as accurately targeting drugs to the appropriate region of the nasal cavity at therapeutically relevant doses, while maintaining reproducibility, make this cutting-edge approach a regulatory challenge. The prolonged path to clinical translation discourages many researchers from pursuing this potentially life-saving strategy. Nevertheless, preclinical studies demonstrate that intranasal delivery can achieve up to ten-fold higher concentrations of select drugs in the brain. Cancer chemotherapeutics span a wide range of molecular formats, from small molecules to 150-kilodalton antibodies. Accordingly, delivery strategies must be carefully matched to the molecular properties of each therapeutic. Here, we focus on the intranasal delivery of small molecule inhibitors using nanomaterial-based platforms, including aerosols, lipids, gold nanoparticles, gels, emulsions, fibers, and their combinations. Ultimately, we hope that intranasal delivery approaches will be translated to provide patients with better therapeutic outcomes.

## 1. Clinical Rationale: Why This Field Exists

### 1.1. Therapy of Brain Tumors Remains Limited by Delivery, Not Just Potency

The average survival after a glioblastoma diagnosis is approximately fourteen months with the current standard of care, which includes surgical removal of the tumor and then ongoing treatment with radiation and oral chemotherapy [1]. There are many difficulties in treating glioblastoma, such as its physical location making tumor resection highly invasive and dangerous as well as the high dosages of therapeutics that may cause systemic toxicity and off-target effects. From a molecular standpoint, glioblastoma is cancer that has a heterogeneous population of cells, meaning that parts of the tumor may have different genetic signatures that require combination therapy.

While the incidence of brain disease has been increasing in recent years, central nervous system (CNS) therapeutics have low success rates due to complex biological, technical, clinical, and regulatory challenges. We have compiled, from recent works in this field, the top eight hurdles (H1–H8), where the challenge will be described, and the following sections suggest possible intranasal nanoparticle solutions that may overcome these hurdles.

### 1.2. Why Nose-to-Brain Delivery Is Attractive

Conventional oral administration commonly does not achieve therapeutic levels in the brain due to the structures of the blood–brain barrier [2]. The blood–brain barrier remains the primary obstacle to effective drug delivery, since its function is to highly regulate and filter which molecules can pass through the central nervous system, thus increasing the requirements for effective therapy. Studies tracking nanoparticles fabricated to cross the blood–brain barrier show that less than 20 percent of the expected drug concentration was found at the target, along with substantial off-target accumulation in preclinical rat trials [3,4].

Nose-to-brain delivery is favorable because it is a noninvasive and repeatable therapy. Noninvasive routes include lipid nanoparticles, polymer-based nanoparticles, cell-derived exosomes, hydrogel delivery systems, micron delivery systems, protein nanoparticles, and inorganic nanoparticles. Common intranasal strategies use direct drug absorption and permeation enhancers, but suffer from poor drug stability, limited availability to the target, and quick clearance [3,5,6,7,8]. New nano delivery methods are being investigated to functionalize nanoparticles for enhanced drug stability, increased nasal residence, and increased mucosal penetration. Recent advances in liposomes, polymeric nanoparticles, and nanogels have been investigated to encapsulate therapeutics and incorporate ligands that would help target tumors, with some emerging trends including AI-driven formulation design [3,5,6,7,8]. Current challenges impede clinical translation, such as safety concerns, manufacturing scalability, and regulatory barriers. These all demonstrate a need for highly targeted and specialized therapeutics.

## 2. Translational Hurdles in Nose-to-Brain Chemotherapy

Although multiple reviews describe individual intranasal delivery technologies, they primarily compare nanomaterial composition, formulation strategies, or biodistribution outcomes. Relatively few reviews evaluate these systems according to the translational barriers that ultimately determine clinical implementation. To address this gap, we organize these interconnected physiological, pharmacokinetic, manufacturing, and regulatory challenges into an eight-component translational hurdle framework (H1–H8). This framework serves as the foundation for the platform comparisons, first-in-human (FIH) readiness assessment, and minimum reporting recommendations developed throughout the remainder of this review.

**Figure 1:**
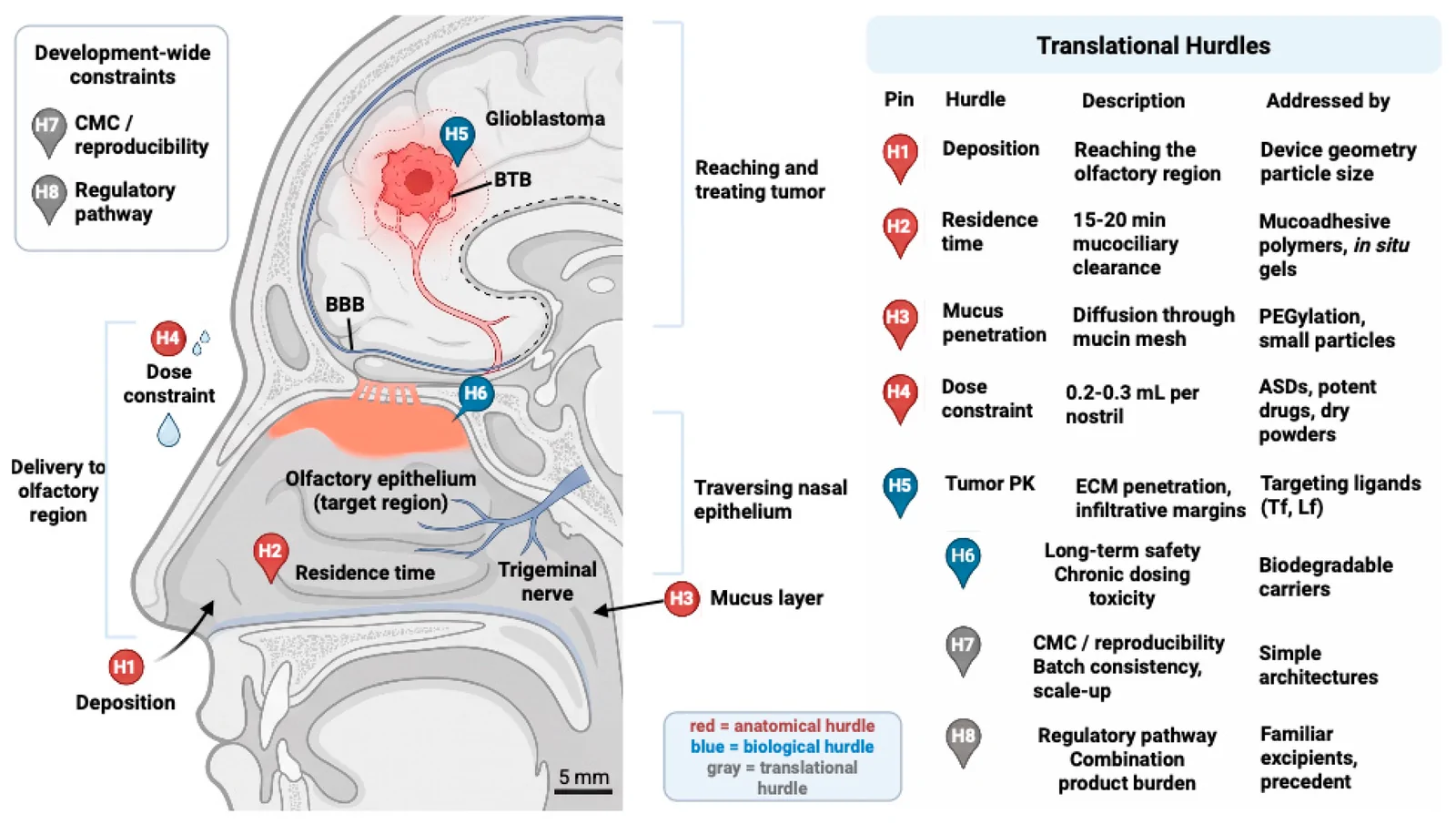
Translational hurdles for intranasal nose-to-brain chemotherapy in glioblastoma. Eight hurdles (H1–H8) are grouped by the stage at which they arise. Anatomical hurdles (H1–H4, red) reflect physical constraints on drug deposition and residence in the nasal cavity; biological hurdles (H5–H6, blue) reflect pharmacokinetic and safety challenges once the drug reaches its target; translational hurdles (H7–H8, gray) apply throughout development and regulatory review. Nanomaterial platforms are evaluated in later sections by which hurdles they address (see Table 1 and Section 5). Created in BioRender. Keniry, M. (2026) https://BioRender.com/ecibxpz.

As shown in Figure 1, these hurdles are categorized chronologically at the stage they arise in: anatomical hurdles (H1–H4) reflect physical constraints within the nasal cavity, biological hurdles (H5–H6) reflect pharmacokinetic and safety challenges at the target site, and translational hurdles (H7–H8) govern manufacturing and regulatory evaluation.

**Table 1:** Summary of nanomaterial delivery platforms for intranasal nose-to-brain chemotherapy of glioblastoma, characterized by structure, mechanism, key advantages and limitations, first-in-human (FIH) readiness, tier classification, and representative manuscript references.

Platform	Structure	Primary Mechanism	Key Advantages	Key Limitations	FIH Readiness	Tier	References
Polymeric nanoparticles PLGA, chitosan	Solid biodegradable polymer core, 100–300 nm	Encapsulation with controlled release; surface modification for mucoadhesion or targeting	Biocompatible; mucoadhesive (chitosan); established CMC precedent; biodegrades to non-toxic byproducts (lactic/glycolic acid)	Mucus binding vs. penetration trade-off; batch variability; limited tumor PK without targeting ligands	High	1	[9,10,11,12]
Polymeric micelles PEGylated, stimulus-responsive	Amphiphilic block copolymer core–shell, 10–100 nm	Self-assembly around hydrophobic drugs; stimulus-triggered release	Excellent for hydrophobic drugs; Genexol-PM clinical precedent for paclitaxel delivery	Dilution instability below CMC; aerosolization stress; limited intranasal precedent	Moderate	2	[13,14,15,16,17,18,19,20,21,22]
Lipid nanoparticles (LNPs) with mucoadhesive fibers	Ionizable lipid + PEG-lipid + cholesterol + helper lipid, 40–200 nm	Endosomal escape; mucoadhesive fiber coating extends residence time	mRNA vaccine precedent (Pfizer/Moderna/Shingrix) provides regulatory pathway; high payload versatility	Cilia damage risk ^a^; intranasal chemotherapy application immature; combination-product regulatory burden with fibers	Moderate	2	[23,24,25,26,27]
Hydrogels in situ gelling systems	3D polymer network; thermosresponsive (poloxamer) or ion-sensitive (chitosan)	Liquid-to-gel transition on administration; sustained release from swollen matrix	Directly addresses residence time (H2) and dose (H4); mucoadhesive; well-tolerated	Restricted diffusion through nasal mucosa (H3); gelation sensitive to pH/temperature; standardization challenges (H7)	High	1	[28,29,30,31,32,33,34,35,36,37,38,39,40,41,42,43,44]
Amorphous solid dispersions ASDs	Drug dispersed in amorphous state within polymer matrix (HPMC-AS, PVP)	“Spring and parachute” solubility enhancement; hydrogen bond crystallization inhibition	Solves H4 (dose constraint) via high loading; strong regulatory precedent; suitable for poorly soluble small molecules	Physical stability during storage; mucus trapping in powder form; recrystallization risk	High	1	[45,46]
Gold nanoparticles AuNPs	Rigid inorganic core with functionalizable surface, 5–100 nm	Surface conjugation of drugs, imaging agents, or targeting ligands; photothermal capability	Precise size and shape control; multifunctional (theranostic potential); rigid structure resists degradation	Chronic accumulation in liver/kidney (H6); non-biodegradable; combination-product regulatory burden (H8); intranasal precedent lacking	Low	3	[47,48,49,50,51,52,53,54,55,56,57,58,59,60]
Ferritin nanocages protein-based targeting	24-subunit self-assembling protein cage; 12 nm outer/8 nm inner	Native TfR1/SCARA5/TIM-2 receptor binding for tumor targeting; drug encapsulation in cage lumen	Excellent tumor PK (H5) via native receptor targeting; up to 4× MTD improvement in preclinical studies ^b^	Immunogenicity risk on repeated dosing (H6); manufacturing heterogeneity (H7); no intranasal precedent (H8)	Low	3	[9,10,11,12,13,14,18,19,61,62,63,64,65,66,67]

Tier assignments correspond to the tier classification framework in Section 5, in which Tier 1 platforms combine high FIH readiness with broad coverage of translational hurdles, Tier 2 platforms are realistic candidates requiring additional development, and Tier 3 platforms face significant safety, manufacturing, or regulatory barriers before near-term clinical translation is viable. References are the manuscript entries that most directly support each platform’s characterization; complete citation details appear in the references section. *FIH readiness reflects the authors’ interpretation of manufacturing simplicity, regulatory precedent, and toxicology track record; placements may shift as new clinical data emerge*. ^a^ Reported in multiple LNP intranasal formulation studies; magnitude depends on lipid composition and dosing frequency. ^b^ Fold-improvement in maximum tolerated dose (MTD) is model-dependent and observed in preclinical GBM studies; clinical validation pending.

While the standard literature often highlights preclinical brain accumulation, these metrics routinely fail to predict human clinical trial success due to unrecognized anatomical gaps and oversimplified assumptions [5,6,7,8]. Identifying and addressing these translational hurdles is important for establishing a reproducible minimum reporting standard to bridge the lab-to-clinic gap in glioblastoma (GBM) therapy.

Every hurdle contributes to translation, but current evidence suggests that they are not equally limiting. Three of the eight hurdles represent the main barriers to clinical implementation: anatomical deposition (H1), dose constraint (H4), and tumor-specific pharmacokinetics (H5). These hurdles determine whether a formulation can reliably reach the appropriate anatomical region in humans, overcome the limitations of preclinical animal models, and achieve therapeutically meaningful drug concentrations within infiltrative glioblastoma tissue. Improvements in residence time, drug loading, manufacturing reproducibility, or regulatory strategy remain important; however, these later-stage optimizations cannot compensate for inadequate delivery to the therapeutic target.

### 2.1. Anatomical Variability and Deposition

**Figure 2:**
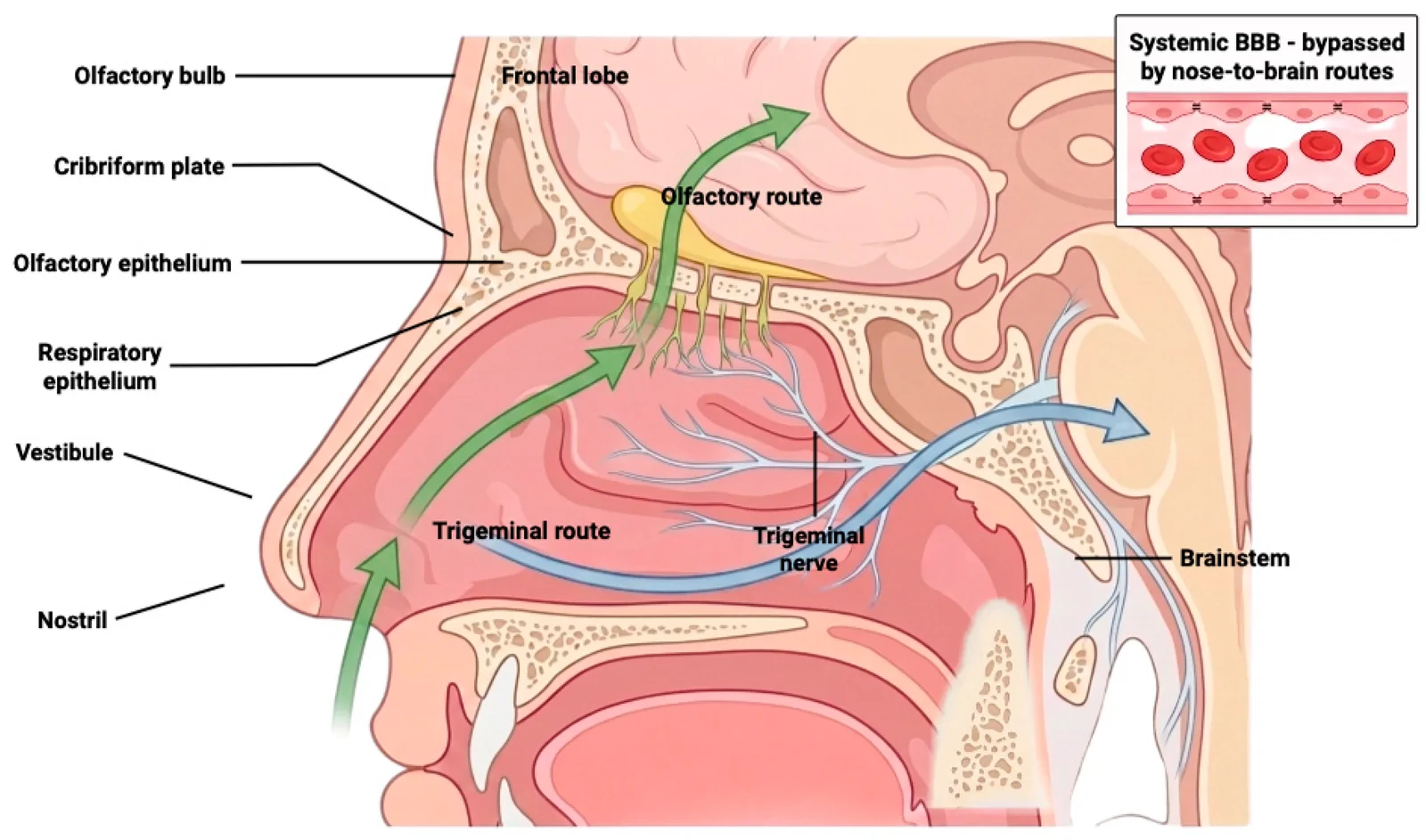
Anatomy of the nasal cavity and nose-to-brain transport routes. Drugs administered intranasally can reach the CNS via the olfactory pathway (green arrow: olfactory epithelium → cribriform plate → olfactory bulb → forebrain) or via the trigeminal pathway (blue arrow: nasal mucosa → trigeminal nerve branches → brainstem). Both routes bypass the systemic blood–brain barrier (BBB, inset). Created in BioRender. Keniry, M. (2026) https://BioRender.com/ei4277r.

Reliable nose-to-brain therapeutic delivery depends on consistent drug deposition in the upper regions of the nasal cavity, specifically the olfactory epithelium, Figure 2 [5,6]. Yet, human nasal anatomy is notoriously variable. Differences in nasal geometry, morphology, airflow dynamics, and the location of the olfactory cleft substantially influence drug distribution, making reproducible targeted delivery difficult in practice [7]. Conventional nasal sprays frequently deposit most of the administered dose within the anterior respiratory region rather than the olfactory epithelium, limiting their therapeutic efficiency [8].

Recent advances in intranasal delivery devices have attempted to address these anatomical limitations. Breath-powered bi-directional exhalation delivery systems utilize patient exhalation to elevate the soft palate and dilate the nasal valve [68]. Other emerging technologies, including precision nasal applicators and patient-specific delivery devices, utilize optimized spray trajectories and computational airflow modeling to improve deposition within the upper posterior nasal cavity and olfactory region [69]. These systems reduce the dependence of deposition on patient technique and nasal anatomy, thereby improving reproducibility. Compared with conventional nasal spray pumps, these approaches demonstrate improved deposition and reduced interpatient variability, partially addressing H1 (target-region deposition), but do not substantially mitigate downstream hurdles including H2 (mucociliary clearance), H5 (tumor pharmacokinetics), or H6 (repeated-dose safety).

Although necessary, improved anatomical targeting alone is insufficient for successful clinical translation. Even when deposition in the olfactory region is achieved, other physiological barriers, including mucociliary clearance, limited dosing volume, tumor-specific pharmacokinetics, and repeated-dose safety, continue to complicate effective delivery.

### 2.2. Mucociliary Clearance and Dose Constraints

Mucociliary clearance is a particularly formidable obstacle. As an innate defense, this process quickly removes foreign particles from the nasal cavity, significantly decreasing the drug residence time and limiting absorption [70,71]. While mucoadhesive materials can extend this residence time, they often create a new problem: a trade-off where excessive adhesion prevents the drug from penetrating the mucus layer [72,73]. In addition to these challenges, the nasal cavity can only accommodate small administration volumes, usually between 0.2 and 0.3 mL per nostril [74]. To be effective, delivery systems must therefore utilize highly potent drugs or achieve exceptionally high drug loading [72].

### 2.3. Preclinical-to-Human Gap

Beyond delivery mechanics, translational challenges are further amplified by discrepancies between preclinical models and human physiology. The gap is largely anatomical. Preclinical success in rodent models often overestimates delivery efficiency in humans, since rodents have a proportionally larger olfactory region [2,75]. This anatomical difference facilitates a more direct transport route that simply does not exist in humans.

Even successful delivery to the brain does not guarantee therapeutic efficacy. Drug presence within the brain alone is insufficient; effective treatment requires adequate drug concentrations within tumor tissue and its infiltrative margins, which are often poorly reached [76,77]. Too many studies focus on whole-brain distribution while neglecting tumor-specific pharmacokinetics, which limits their translational relevance.

### 2.4. Long-Term Safety and Manufacturing

Safety concerns also grow with repeated administration. Chronic dosing may lead to local toxicity issues, including epithelial damage, irritation, and inflammation [78]. Moreover, the impact of long-term exposure to nanomaterials raises concerns regarding central nervous system safety, particularly under repeated dosing regimens [79].

In parallel, successful clinical translation requires extensive manufacturing and quality control. Nanomaterial systems must demonstrate reproducibility in parameters including particle size, surface charge, drug loading, and release behavior [80,81]. Stability during storage and administration is also very important, as variability can significantly impact both the efficacy and safety of the drug delivery system [82,83].

### 2.5. Regulatory Complexity

These technical challenges are further compounded by regulatory complexities. Intranasal nanomedicines are often classified as combination products, which require evaluation of both the drug formulation and the delivery device [84,85]. This increases the regulatory burden and adds a layer of required comprehensive safety and performance data [86]. Ultimately, clinical adoption depends on demonstrating clear advantages over existing standards of care, including systemic chemotherapy and alternative delivery routes [87]. Without meaningful comparative benefits, successful translation to clinical practice remains very unlikely [88].

### 2.6. Intranasal Deposition Barrier for Accurate Cancer Chemotherapeutic Dosing

Aerosols are commonly used for the nasal delivery of anti-inflammatories (e.g., fluticasone, mometasone) [89]. This method is familiar and scalable, with the possibility of repeated dosing. Aerosols have been examined for the ability to deliver cancer chemotherapeutics to the brain via the nasal cavity (e.g., NEO100, which has begun Phase I and II clinical trials) [90]. However, in practical terms, there is tremendous human variability with using nasal sprays (e.g., a computational study across four ethnic groups found wide variation in nasal proportions and spray uptake) [91]. Another hurdle is that low-viscosity aerosols are quickly cleared from the nasal cavity. Targeting the olfactory nerve region of the nasal cavity reproducibly may prove challenging, in part because sprays can vary widely in droplet size and content (particles below ~10 µm tend to agglomerate owing to their high surface area, whereas larger particles deposit preferentially in the anterior nasal passage) [92]. The variability aspect of aerosols makes this method almost unfeasible, as variations in cancer chemotherapeutic drug dosing are not acceptable.

Powders such as amorphous solid dispersions (ASDs) hold promise as a chemotherapy delivery method due to greater drug dosing, greater stability and longer resistance in the nasal cavity. Many small molecule inhibitors are poorly soluble in aqueous solutions such as those found in aerosols but can be embedded into powders at much higher dosages [93]. Although the residence time of ASDs is typically far greater than aerosols, the powder can get trapped in mucus, leading to inconsistent drug delivery.

Hydrogels are much more viscous than aerosols, leading to greater nasal retention and a much higher likelihood of chemotherapeutics reaching the appropriate area of the nasal cavity for delivery to the brain. However, the issue of mucus trapping remains, and the problem of drug degradation is greater compared to powders with increased molecular mobility and the presence of water in hydrogels. There is an added issue: even if the drug is stably delivered to the appropriate area of the nasal cavity, will it reproducibly transit the nerves/intercellular spaces cross into the brain to reach the tumors? More testing is needed to evaluate intranasal drug delivery efficiency and reproducibility using highly promising hydrogels before their clinical deployment.

Fibers embedded with chemotherapeutics can be placed as an insert into the nasal cavity near the trigeminal and olfactory nerves. For example, Propel is an FDA-approved sinus implant that delivers steroids to target inflammation [94]. This nasal insert underwent several clinical trials that demonstrated improved patient outcomes following sinus surgery. Fibers can be fabricated from an array of materials, including polycaprolactone (PCL), chitosan (CS), polyvinyl alcohol (PVA), poly(lactic-co-glycolic acid) (PLGA), polyvinylpyrrolidone (PVP) [95], and Nylon-6. These inserts are nasally placed in an outpatient setting and biodegrade within two months. This leads to sustained drug delivery that can be repeated. However, the drugs that are combined with the fibers must be stable and not cause irritation.

## 3. Nanomaterial Platforms

A wide range of nanomaterial-based delivery systems have been explored to improve the efficiency of intranasal drug delivery for brain tumors. While many nanomaterials demonstrate promising clinical performance, their translational success depends not only on their ability to enhance drug delivery, but also on their stability, manufacturability, and regulatory feasibility.

Each nanomaterial platform offers specific advantages based on its physicochemical properties and design strategy. The clinical potential of these platforms is best evaluated by how effectively they address existing hurdles without creating significant new ones. In this section, we assess major nanomaterial systems based on their ability to overcome key translational challenges, as well as their first-in-human (FIH) readiness.

### 3.1. Polymeric Nanoparticles

Polymeric nanoparticles, specifically those composed of biodegradable polymers such as poly(lactic-co-glycolic acid) (PLGA), are one of the most extensively studied nanomaterial platforms for drug delivery [96,97]. These systems are designed to protect encapsulated drugs from degradation while enabling controlled and sustained drug release, which makes them especially attractive for applications in brain tumor therapy [98]. In addition to PLGA, other polymeric systems like chitosan, alginate, and gelatin-based nanoparticles have also been explored because of their biocompatibility and tunable physical and chemical properties, allowing for the modification of interactions with the nasal mucus layer and epithelial tissues [99,100].

One of the main advantages of polymeric nanoparticles is their ability to enhance nasal residence time through mucoadhesive surface modifications [101]. For example, chitosan-based nanoparticles have been studied for their inherent mucoadhesive properties [45], which promote longer interaction of the drug with the nasal mucosa and improve drug retention [102]. Surface modification strategies such as PEGylation have also been used to reduce mucus binding and improve particle diffusion [103]. This process may reduce retention time, which highlights the trade-off between mucus penetration and adhesion [104]. Polymeric systems benefit from more well-established manufacturing processes that support their reproducibility and quality control compared to other complex nanomaterial systems [105]. These systems can achieve high drug loading, making them suitable for addressing dose limitations associated with the nasal cavity’s small volume [106]. The use of biocompatible and clinically familiar polymers often results in manageable safety profiles, which enhances patient tolerance for repeated dosing.

Despite these advantages, nanoparticles do not fully overcome translational challenges. Deposition remains largely dependent on the delivery device instead of the nanoparticle itself, meaning that even well-designed systems may fail to consistently reach the olfactory region [107]. Also, while polymeric nanoparticles can improve drug stability and residence time, there is limited evidence of adequate tumor and margin distribution that would lead to any meaningful tumor pharmacokinetics [108]. Targeting strategies, such as ligand-functionalized nanoparticles (e.g., transferrin or lactoferrin conjugated systems [106]), have been explored to enhance tumor uptake. However, these approaches require robust validation in human-relevant models [9,10]. Strategies aimed at increasing cohesion can also reduce penetration through the mucus membranes.

### 3.2. Polymeric Micelles (PEGylated and Stimulus-Responsive Variants)

Current functionalized nanoparticles (FNPs) are usually made in organic phases, which results in hydrophobic surfaces. This does not work well for biomedical applications since the nanoparticles would need to be water-dispersible and biocompatible. Techniques such as ligand exchange and surface modification could be used to solve this problem, but polymer micelle encapsulation provides a solution that does not depend on functional groups, which typical FNPs usually lack [23]. Micelles have a core–shell structure that would encapsulate the hydrophobic FNP. Self-assembling polymer micelles are fabricated in an aqueous solution by amphiphilic copolymers and produce a hydrophobic core and hydrophilic shell that stabilizes the nanoparticle [109]. Hydrophilic and hydrophobic components are as follows.

Polyethylene glycol (PEG) is commonly used for the hydrophilic segments. It increases water solubility and biocompatibility, which promote circulation time and add more functional groups for further surface modification. PEG has an abundance of hydroxyl groups which offer excellent water solubility, strong biocompatibility, and lack of toxicity, along with solid physical stability and prolonged biological activity. Other common hydrophilic polymers are methoxy PEG- and acid-sensitive poly(2-ethyl-2-oxazoline) [110].

Hydrophobic components commonly used are PCL, PLGA, poly(benzyl aspartate), and PLA [111]. PLA is a highly researched polymer due to its sustainable, biocompatible, and biodegradable properties [112,113]. PLGA is also highly researched, has FDA approval, and is a beneficial polymer because, in addition to the properties of PLA, PLGA also adds mechanical strength [113,114]. An emerging area of research in hydrophobic components is polyamino acids such as polyglutamic acid, polylysine, and polyaspartic acid [115,116]. These are useful because they have extremely reactive carboxyl and amino groups which degrade into amino acid monomers and easily integrate hydrazone bonds, phenylimine bonds, and acetals [116,117]. Polylysine is most promising in gene carrier materials due to its free amino acids and its ability to dissociate into cationic ions that bond with anionic nucleic acids via robust electrostatic interactions [118]. These can be optimized for encapsulation efficiency, loading capacity, and delivery efficacy and further modified as stimulus-responsive polymeric micelles to enhance in vivo stability, biocompatibility, and targeted delivery [119].

#### 3.2.1. Formation Techniques

There are three main techniques used in forming the nanoparticles for PEGylation, which all have certain strengths based on formulation [120]. Physical embedding is solubilizing drugs in polymer micelles via hydrophobic interactions and hydrogen bonds via emulsion, solid dispersion, or dialysis [120,121]. Chemical binding is based on covalent bonding of the drug molecules onto hydrophobic polymer chains [121]. These types of formulation avoid dilution once the nanoparticles have entered the bloodstream and thus increase bioavailability [121]. Since the micelles fabricated using this method are formed by strong covalent bonds, they can carry high drug load. The caveat is that these bonds are formed through electrostatic interaction and thus need the appropriate charges [119]. The modular chemistry of this type of polymeric micelle would assist with combination loading as well as potentially improve H5 (pharmacokinetics) by functionalizing the micelle to target specific aspects of the tumor [121].

#### 3.2.2. Stability Challenges

Challenges for polymer micelles are the structural changes faced from pH, temperature, dilution, and ionic strength. Without optimization to correct these issues, premature release of the drug may occur [122]. This is where stimulus-responsive micelles are advantageous since they are dependent on specific stimuli for drug release and can be modified to react to physiological factors, chemical factors, or physical factors [119]. Crosslinking does increase stability and can be achieved through ionic bonds in the hydrophilic shell and the hydrophobic core, as well as the core/shell interactions [123]. For example, core-crosslinked micelle designs can support high drug loading (on the order of 50% by weight) while resisting dilution [123,124]. Photoinduced Bergman cyclization polymerization was used to crosslink the enediyne (EDY) hydrophobic segments of amphiphilic EDY–gemcitabine (GEM) conjugates, in which GEM served as the hydrophilic component, producing micelles with drug loading of up to 50% by weight [124]. In this case, core-crosslinked nanoparticles compared to non-crosslinked were more stable under dilution. Drug release is also lower at neutral pH for core crosslinking [124]. Another study used PEG-PMPC-fabricated micelles that were small, maintained stability during dilution and had the capability to encapsulate DOX [125]. Polymeric micelles solve the issue of solubilization of hydrophobic materials helping H3 (mucus penetration) due to its high apparent solubility. If the micelle is also paired with a mild mucoadhesive or gel, this could also increase H2 (residence) by increasing the time the drug would stay in the nasal passage. Challenges pertaining to H7 (quality control and storage) do bring a small setback due to dissociation and dilution stability but with surfactants, as long as they do not interfere with H6 (irritation) and can prove to have moderate FIH realism.

### 3.3. Lipid Nanoparticles (LNPs) and LNP-Loaded Mucoadhesive Fibers

What they solve best: potent encapsulation for certain payload classes and manufacturing precedent in other indications.

#### 3.3.1. LNPs with Mucoadhesive Properties

Lipid nanoparticles are FDA-approved in therapeutics, such as the Pfizer and Moderna COVID-19 mRNA vaccines and GSK shingles vaccine Shingrix [126]. Current LNP formulations are made with ionizable lipids, which retain a near-neutral pH 7.4 (while in circulation) and are better tolerated than cationic formulations [127]. In acidic environments (such as in endosomes), ionizable lipids are protonated, leading to a lower pH of 5–6.5 and therefore endosomal escape, allowing for payload delivery [127,128]. This pH-responsive behavior is governed by the lipid’s pKa and is arguably the most critical parameter when designing LNPs [127]. LNPs most commonly have a hydrophilic aqueous core, which is ideal for nucleic acids [129]. However, some LNPs have a solid core that can entrap hydrophobic molecules [129]. In the context of cancer, ionizable LNPs can be amenable to repeated dosing, given the lower toxicity, and can accommodate many different types of cargo such as nucleic acids and small molecules [130]. The strengths with using LNPs include the potential for high levels of payload and increased cellular uptake of reagents [129].

#### 3.3.2. Solid Lipid Nanoparticles (SLNs) and Nanostructured Lipid Carriers (NLCs)

Both solid lipid nanoparticles (SLNs) and nanostructured lipid carriers (NLCs) are structurally different, and yet are related classes of lipid-based platforms that differ from LNPs primarily by using a solid or semi-solid lipid matrix rather than an aqueous or ionizable lipid core. SLNs that are considered to be first-generation are entirely composed of solid lipids that remain solid at both room and body temperature, whereas NLCs that are considered to be second-generation combine solid and liquid lipids (a 70:30 ratio) to improve drug loading capacity and reduce drug expulsion during storage [131]. Due to the ability of SLNs to remain solid at both body and room temperature, this poses a caveat that limits drug loading capacity, since the crystalline lipid lattice leaves little space for drug incorporation [132]. Both SLNs and NLCs rely on their small particle size (<1000 nm) and their lipophilicity to penetrate the nasal mucus layer and achieve a rapid uptake by olfactory and trigeminal neurons, facilitating nose-to-brain transport [132]. In vivo studies report measurable brain accumulation within minutes of intranasal administration, with some studies reporting a time to maximum brain concentration as little as 10–30 min after administration [133,134]. Within the context of GBM, both SLNs and NLCs have been explored extensively as brain-targeting carriers, including chitosan-coated ones that are designed to enhance intranasal residence time and tumor targeting [135]. Recently, NLCs have made significant progress towards clinical translation through the implementation of temozolomide conjugated with anti-PD-L1 single-chain variable fragments for combined chemo-immunotherapies [136]. These NLCs were able to cross the BBB and resulted in stabilized tumor growth beginning in the third week of treatment onward compared to free drug or untargeted nanoparticle controls in an orthotopic glioma model, highlighting this platform’s potential for clinical translation, especially when considering combination GBM therapies [136]. However, storage stability remains a critical point of difference between SLNs and NLCs. Since SLNs are composed of a single and highly ordered solid lipid matrix, they are prone to lipid polymorphic transitions during storage that may expel the encapsulated drug over time [136]. NLCs, on the other hand, mitigate this issue by incorporating the use of a liquid lipid fraction into the solid lipid matrix that can disrupt crystalline order, lower the risk of drug expulsion and improve both the drug retention and long-term storage stability [136].

#### 3.3.3. Polymeric Micelle-Incorporated Fibers (And Other Fiber-Based Hybrids)

Polymeric micelles are particularly useful for hydrophobic drug delivery and are already FDA-approved in the instance of Genexol, which assists with paclitaxel drug delivery for breast cancer [23]. Polymeric micelles are nanoscopic (10–100 nm) core–shell structures formed by the self-assembly of amphiphilic block copolymers in an aqueous solution [137]. They consist of a hydrophobic core that solubilizes poorly soluble drugs and a hydrophilic shell (or corona) that stabilizes the structure in water, typically formed from PEG [137]. These drug-loaded polymeric micelles can be spun into fibers such as PLGA or PVDF using electrospinning or Forcespinning to increase their stability [95,138]. Embedding micelles into the nanofibers also promotes the proper placement of the drug within the nasal cavity, with a potential for controlled release [138,139].

#### 3.3.4. Exosomes

Exosomes are naturally occurring phospholipid membranous carriers that have proteins embedded in them [140,141,142]. These membranes carry proteins and nucleic acids to other cells [140,142,143]. Although no current therapy that employs exosomes has been FDA-approved yet [144,145], this naturally occurring delivery system has tremendous biocompatibility and potential as an intranasal delivery vehicle [146,147]. Researchers purify exosomes from many sources, including curated cell lines [148], bovine milk [149,150] and even low-cost food sources such as grapefruit [151,152], and can then embed within them small molecule inhibitors (such as doxorubicin or paclitaxel) [152,153,154] or nucleic acid inhibitors such as RNAi [150,155]. The stability of exosomes is only 24–28 h at room temperature [156,157]. IV exosomes are quickly cleared from the blood by the liver (2–30 min in rodents) [158,159,160], similar to LNPs [161].

### 3.4. Hydrogels (Including Nanoparticle-in-Gel Hybrids)

The hydrogel-based delivery system represents a practical strategy that is often used for mucociliary clearance (H2) [28,29,30,162]. Typically characterized by high biocompatibility, hydrogels are hydrophilic, three-dimensional polymer networks that can retain large amounts of water while maintaining their structural integrity [30,31,32,33]. In intranasal delivery, in situ gelling systems, such as thermoresponsive (e.g., poloxamers) and ion-sensitive (e.g., chitosan) polymers allow for liquid-to-gel transitions upon their administration [34]. This phase transition allows for improved retention at the nasal epithelium and prolonged drug exposure, directly addressing the challenge of insufficient residence time, which often limits drug absorption and therapeutic efficacy in GBM treatments [34,163].

One advantage of hydrogel systems is their ability to enhance nasal residence time (H2) and their ability to sustain drug release [28]. Through mucoadhesion to the nasal epithelium, hydrogels reduce rapid clearance of intranasal drug administration and maintain the drug concentration gradient, which favors transportation along the trigeminal and olfactory pathways [28]. Several preliminary studies have shown that hydrogel-based systems significantly increase drug retention and enhance delivery to the brain compared to conventional liquid systems [35,36]. Additionally, hydrogels can inhibit enzymatic degradation of encapsulated drug treatments within the nasal cavity, thus improving drug stability and bioavailability [28]. From an engineering perspective, polymer composition, gelation temperature, viscosity, and crosslinking density need to be optimized to ensure effective nasal administration and sufficient in situ gel formation [37]. When these systems are combined with nanoparticles, they allow for the integration of controlled drug release with enhanced uptake and targeting potential, offering a multifunctional approach to intranasal delivery [38].

Utilizing hydrogels has drawbacks, such as the crosslinking density, and polymer composition can significantly limit the penetration of larger molecules [37]. In addition to this limitation, the variability of hydrogel formulation poses a great challenge for ensuring quality control (H7). The gelation behavior, viscosity, and rheological properties are known to be highly sensitive to changes in environmental conditions such as pH, temperature, and ionic strength [39,40]. These changes can severely complicate manufacturing standardization. From a safety perspective (H6), repeated intranasal exposure to certain polymers can lead to irritation and epithelial damage, which necessitates thorough evaluation of efficacy and safety.

The storage and manufacturing stability of in situ nasal gels, including hydrogels and nanoparticle-in-gel hybrids, remains a significant translational concern due to the lack of long-term testing in practical applications. Attributes such as viscosity, mucoadhesive strength, and gelation temperature are susceptible to deviating from acceptable standards during storage, which can compromise the performance of the product and, in turn, patient outcomes. These concerns regarding stability are further intensified by sterilization requirements and manufacturing standards. Following these requirements and standards may alter the crosslinking density of the gel matrix and may introduce variability between products [164].

In comparison to other nanocarrier platforms that address similar translational hurdles, hydrogels primarily rely on mucoadhesion and viscosity to prolong nasal residence time, whereas lipid-based nanocarriers such as NLCs and SLNs primarily rely on their small size and high lipophilicity to permeate the mucus layer and allow diffusion through the mucus to enhance drug delivery [131,133]. In vivo studies have indicated that NLCs and SLNs can achieve measurable accumulation within the brain within minutes of intranasal administration, which is an advantage over the slower and limited diffusion release from mucoadhesive hydrogels. However, hydrogels provide substantially longer nasal residence time and sustained release, as well as avoiding lipid oxidation instability, which can affect NLC or SLNs during storage [131,133]. If these two platforms were to be combined—for example, by embedding an NLC or SLN within an in situ gelling hydrogel matrix—this approach could offer the rapid mucosal permeability of lipid nanocarriers along with an extended residence time [38].

Despite the expanding sophistication of gel formulations and integrating nanocarriers to enhance drug delivery, there remains a notable lack of systematic studies that evaluate the effects of storage, sterilization, and manufacturing processes on both the functional and physicochemical properties of these systems [164]. Although some studies have reported the use of stabilizing additives, such as preservatives like benzalkonium chloride, and solubility enhancers like cyclodextrins, their impact on the long-term storage and manufacturing stability is not comprehensively addressed. This gap remains pronounced for complex hybrid systems, including hydrogels and nanoparticle-in-gel hybrids, and highlights the need for maintenance on both the integrity of the gel matrix and the stability of the embedded nanoparticles within these systems. Thus, systematic evaluation of the shelf-life, freeze–thaw stability, and reproducibility within these systems is necessary before hydrogel-based platforms can transition into large-scale clinical supply.

When considering a translational perspective, hydrogel systems exhibit high to moderate first-in-human (FIH) readiness, especially when these systems are formulated using biocompatible polymers that have predictable gelation mechanisms [40]. When compared to complex nanoparticles, hydrogels are reliable candidates for clinical development due to their ease of manufacturing and established regulations, especially when using polymers that have prior clinical use (e.g., chitosan derivatives) [38,41]. The translational potential of hydrogels has been shown to be the strongest when the system is supported with pharmacokinetic data, which demonstrates improved drug distributions to tumor regions [46]. Due to these characteristics, hydrogels provide a balance between practical applications and enhanced delivery, making them a promising strategy for clinical translation in intranasal GBM therapies [35,46].

### 3.5. Amorphous Solid Dispersions (ASDs)

Amorphous solid dispersions increase solubility and bioavailability of poorly water-soluble drugs. Up to 40% of marketed drugs and 90% of pipeline drugs are poorly soluble. ASD has an active drug dispersed in amorphous form within a polymer matrix. The amorphous state requires no energy to break the crystal lattice of the molecule, which increases the apparent solubility. There are many techniques as it is a growing field for drug delivery. For the formulation to be efficient, physical stability must be achieved. Some of the barriers that prevent physical stability include formulation, equipment, process variables, and downstream processing. There are a few different techniques employed, such as spray drying, electrospraying, and rotary evaporation [165].

#### 3.5.1. Physical Stability

Amorphous phase separation (APS) reduces stability, and occurs where the polymer-rich phase separates from the drug-rich phase. Crystallization is affected by formulation, environmental factors, and processing. These lead to nucleation and crystal growth and are highly affected by temperature, moisture, and mechanical stress. Higher polymer concentration can kinetically stabilize ASD but will not prevent phase separation. Hydrogen bonding in the drug–polymer interaction can lower the chemical potential and reduce the tendency to form a lower energy ordered crystalline structure [166].

Polymers that have a high glass transition temperature (Tg) increase kinetic barriers [93]. Polymers usually follow a phenomenon where molecular mobility becomes negligible at 50 °C below Tg. Common polymers are PVP, PVP/PVA, HPMC, HPMCAS, and Eudragit series [93,165]. Surfactants and additives are used to improve solubility but could also increase nucleation and crystallization. Environmental factors such as moisture, temperature, and mechanical stress during processing and storage matter as well. Moisture, Tg, and increase in molecular mobility promote crystallization and decrease the amorphous stability. This method works by breaking the crystal lattice of the drug and quickly treating it to attain the amorphous state. Other methods include melting the polymer–drug mixture and rapid solidification. Key considerations for solvent evaporation include solvent choice, evaporation rate, residual solvent levels, and environmental impact. Common solvents are acetone, dichloromethane, ethanol, methanol, ethyl acetate, and water. Class III solvents are preferred as residual solvent levels must comply with guidelines [165].

The olfactory and trigeminal regions only make up 5–7% of the epithelium in the nasal passage [72], which is why delivery systems primarily leave particles in the anterior and respiratory regions. This leads to mucociliary clearance where the formulation is eliminated before the drug reaches the brain [71]. Challenges such as attaining specific rheological properties when atomizing formulations into mist can lead to excess liquid dropping out of the nasal cavity or throat deposition [92]. Physical and chemical stability could be affected due to the moisture in the nasal cavity shifting the formulation to favor recrystallization or phase separation, which may allow the drug to precipitate out of solution and lower bioavailability. The nanoparticles must be small enough to not be trapped in nasal mucus in the sub 150–200 nm range. The appropriate surface charge also needs to be attained for this route [99].

#### 3.5.2. FIH Realism: HIGH–MODERATE

ASDs are effective for poorly water-soluble BCS Class II and IV drugs [167] and usually generate a “spring and parachute” mechanism which temporarily induces a highly concentrated and supersaturated state [168]. This does increase oral absorption, proved mathematically by the area under the curve [169]. There is difficulty translating these therapeutics from preclinical to human models because of inherent stability concerns of the amorphous form of the drug [170], although it does allow for concentrated dosing to help with H3 (mucus penetration). The solid state of the material does lend itself well to addressing H7 (characterization) [171]. The drawbacks of ASDs may have challenges, due to H1 (deposition); as it is a powder, there may be issues with H8 (the delivery device) [172]. Since the challenges presented for ASD are similar across all types of nanoparticles (tolerability, device testing, deposition), the benefits of high drug load concentration, stability of the solid state and characterization are justification for the high to moderate first-in-human realism.

### 3.6. Gold Nanoparticles (AuNPs)

As a unique class of nanoparticles, gold nanoparticles (AuNPs) are characterized by high physicochemical stability, malleable surface chemistry, and imaging capability, and have been explored for intranasal drug delivery [47,48]. Due to their inorganic nature, the size, shape, and surface functionalization of AuNPs can be engineered precisely to fit special accommodations, allowing for reproducible and stable formulations [47,48]. These characteristics make AuNPs especially attractive for theranostic applications, which combine both drug delivery systems and imaging into a single platform [47,48,49]. Within the GBM context, AuNPs have been investigated regarding their abilities to enhance drug stability, biodistribution, and targeted delivery through ligand conjugations [42,50,51].

One of the main advantages of AuNP systems is their rigid structure and degradation resistance, which allows them to exhibit consistent physicochemical properties, such as particle size distribution and surface charge [47]. These characteristics are critical for ensuring reproducible research, enabling stable formulations, and meeting regulatory standards (H7). Additionally, interaction with the nasal epithelium can be enhanced by functionalizing AuNPs with targeting ligands, polymers, and/or peptides [52]. Furthermore, their ability to be tracked in real time via computed tomography or photoacoustic imaging is due in part to their optical and electronic properties [53,54]. This directly supports the goal of improving the evaluation of tumor pharmacokinetics (H5) and biodistribution [54,55]. The ability of AuNPs to be tracked is a key advantage in comparison to many organic nanocarriers, which may often lack imaging functionality.

Direct evidence has been established of glioma-specific targeting by using 3 nm glutathione-coated AuNPs. These coated AuNPs accumulated in orthotopic glioma tissue at a rate that was reported to be 2.3× higher compared to surrounding normal tissue. Additionally, the tumor-to-normal brain ratio was reported to be about 12.0, which is 3× higher than that of larger 18 nm AuNPs [173]. These findings were confirmed by both inductively coupled plasma mass spectrometry quantification and fluorescence imaging. Furthermore, renal clearance and redistribution were successfully achieved using these 3 nm AuNPs, which were excreted into the urine with a 52.5% efficiency rate of the injected dose within a 24 h window. Accumulation in the liver and spleen was reported to be very low, with <3% of the injected dose per gram of tissue in major organs [173]. In contrast, larger 18 nm AuNPs were reported to accumulate heavily in the liver and spleen, with 70.7% and 25% of the injected dose per gram of tissue 24 h post-injection, respectively, and were not effectively cleared by the kidneys, with only 1.6% of the injected dose expelled after 24 h [173].

However, despite these advantages, there are significant translational challenges which limit the ability of AuNPs to reach clinical standards [56]. One major concern is long-term safety (H6), as inorganic particles are not typically biodegradable, which raises the concern of accumulation due to repeated administration [56]. Some studies have shown that the size of the nanoparticle, its surface chemistry, and the dose influence biodistribution and clearance within the mucosal membranes, with smaller nanoparticles showing a greater distribution and potential off-target accumulation [57,58]. AuNPs can remain in biological systems and require extensive safety regulations and long-term toxicity studies [174]. These safety regulations are then further complicated by the need to establish solid impurity control and clearance (H7); the smallest variation in synthesis can greatly affect particle behavior and biological interactions [174]. Thus, these factors increase the difficulty of manufacturing and gaining approval compared to simpler delivery systems.

From a clinical perspective, there is a significant risk–benefit challenge (H6, H8) when considering AuNPs. Despite their ability to offer advanced functionalities such as imaging and targeting systems, these abilities must be considered against increased safety burdens and development complexities [54]. In several cases, simpler delivery systems such as polymeric nanoparticles or hydrogels can achieve sufficient therapeutic outcomes with fewer translational barriers compared to AuNPs [37,88]. Thus, as a result, AuNP systems must be justified by clear and substantial improvements in therapeutic efficacy or diagnostic capability.

Recent studies have continued to encourage AuNP-based platforms towards clinical translation using clinically relevant protocols and radiation energies for glioma therapy. A study published in 2025 developed protein-protected ultrasmall gold nanoclusters which were conjugated to a photosensitizer and a cell-penetrating peptide, which were then combined with ultralow dose x-ray induced photodynamic therapy [175]. These nanoclusters were designed specifically to cross the BBB and accumulate in glioma tumors. Results demonstrated that these gold nanoclusters efficiently suppressed orthotopic glioma growth and increased the survival in both mouse and rat models [175]. There was an observed decrease in tumor bioluminescence signals, indicating inhibition of tumor growth, and confirmed inhibition of tumor growth within histological and MRI analysis of mice models [175]. Rat models showed smaller tumor volumes and a sharp decrease in tumor bioluminescence compared to controls after a single intravenous injection. Survival analysis resulted in 38.5% of rats in the treatment group surviving 81 days, whereas control rats died within 23 days [175]. Within both mouse and rat models, the inhibition of tumor growth was confirmed by bioluminescence imaging, MRI, and histological analysis, and survival curves showed the significant extension of survival in treated groups compared to controls. Within this study, a total dose of 2 gray was used with megavoltage x-ray irradiation, which was compatible with clinical radiotherapy protocols [175]. Additionally, nanocluster clearance through renal and hepatic pathways was observed without toxicity. Biodistribution studied showed a high accumulation of the nanoclusters in the liver and kidney with elimination through renal and hepatic excretion. Additionally, blood tests and histological analysis over the course of 60 days showed no observed toxicity in healthy mice and rats [175]. Broader translational reviews of renally clearable AuNPs have emphasized that a reduction in particle size that is below the renal filtration threshold, around 5.5 to 8 nm, is one of the more validated strategies to balance imaging and targeting properties while ensuring long-term safety and stability [176]. By reducing particle size, AuNPs can be efficiently cleared by the kidneys and minimize accumulation within the liver and spleen, thus reducing potential toxicity [176]. Unlike hydrogels or lipid-based nanocarriers that are composed of biodegradable and regulatorily validated inactive ingredients, AuNPs consist of a non-biodegradable inorganic core, meaning that their clinical translation depends more on optimizing a favorable long-term biodistribution and clearance [56,173].

Overall, when taken into consideration, AuNPs demonstrate low FIH readiness for intranasal oncology applications [54]. Clinical translation is most likely to be dependent upon the development of strategies that address and overcome long-term safety and clearance concerns, such as particle designs, biodegradable coatings, or even hybrid systems that easily facilitate elimination [56,57]. Thus, AuNPs are most likely to remain secondary to simpler, more clinically ready delivery systems for intranasal GBM therapy, unless they are supported by strong safety data and/or compelling therapeutic and diagnostic advantages [55,177].

### 3.7. Targeted Protein-Based Nanomaterials (Ferritin Nanocages)

Targeted protein-based nanomaterials, such as ferritin nanocages, represent a biologically driven approach to drug delivery that differs from traditional synthetic nanoparticle systems [61]. Protein cages afford fine precision through genetic engineering at distinct locations, which is not typically possible with the surfaces of synthetic particles [62]. Ferritin is a naturally occurring iron storage protein that self-assembles into nanoscale cage-like structures, which makes it a promising platform for encapsulating and delivering drugs [61,63]. Unlike traditional nanocarriers that depend on passive transport systems, ferritin-based systems offer the potential for biological targeting, specifically through receptor-mediated uptake pathways such as transferrin receptor 1 (TfR1), SCARA5, and TIM-2 [9,64,65].

One of the main advantages of ferritin nanocages is that they have the potential to improve tumor pharmacokinetics by using selective targeting mechanisms. Many tumor cells, including glioblastoma, overexpress specific receptors such as transferrin receptor 1, a characteristic that can be exploited and targeted by ferritin-based systems [10,11]. In one study, H-ferritin-caged doxorubicin demonstrated more than a 10-fold higher drug concentration inside of the tumor compared to free doxorubicin after a single injection [11]. Because ferritin uses receptor-mediated endocytosis instead of just passive movement, it accumulates more precisely in tumors while avoiding healthy organs, which can improve the maximum tolerated dose by up to fourfold, as seen in recent studies [12]. This receptor-driven approach allows for more precise delivery to the tumor tissue and its infiltrative margins, which addresses a large limitation of traditional intranasal delivery systems [13].

Ferritin-based systems also introduce some significant translational challenges. Manufacturing and quality control are very complex compared to synthetic polymer systems, as protein-based nanomaterials require biological-grade consistency and are very sensitive to variations between particles in production and purification processes [14,15,16]. Also, repeated dosing raises concerns regarding immunogenicity and long-term safety, as protein-based carriers may trigger an immune response over time [17,18]. Regulatory complexity also becomes an issue, since these systems can fall under both biological and combination-product pathways, requiring more extensive safety data and its validation [19,20]. Variability in receptor expression across patients and tumor types also introduces uncertainty, since targeting efficiency might not be consistent in large clinical populations [21,22]. Even though their biologically driven targeting strategy is very promising, their clinical success depends on the validation of receptor targets in human tumors as well as the ability to lower immunogenicity and ensure consistent manufacturing. So, ferritin-based systems are usually more likely to succeed in scenarios where strong biological justification and extensive preclinical data support their use.

## 4. Minimum Reporting Framework for Preclinical Studies

Successful clinical translation of an intranasal nanomaterial system depends on innovative design, but also on the quality and relevance of its preclinical evidence. Although many studies demonstrate promising results, inconsistent experimental design and incomplete reporting often limit their translational value. So, establishing a minimum reporting framework is essential to ensure that preclinical findings are both reproducible and clinically meaningful.

### 4.1. Verifying Drug Deposition

One of the most important requirements is to verify drug deposition in the relevant region in the nose. Instead of assuming sufficient delivery to the olfactory region, studies should directly show deposition using imaging or anatomical validation techniques, such as gamma scintigraphy [24], PET, SPECT, MRI, or fluorescence imaging techniques. Without this validation, conclusions about nose-to-brain drug transport remain uncertain.

### 4.2. Repeated-Dose Nasal Tolerability and Disease Models

In addition to deposition, repeated-dose nasal tolerability must be evaluated extensively. Chronic administration studies should include histological analysis of nasal tissue to assess potential epithelial damage, inflammation, or long-term toxicity. This is very important for nanomaterial-based systems, where cumulative exposure can introduce safety concerns. In addition, it is important to use appropriate disease models for drug delivery. Orthotopic tumor models, which more accurately replicate tumor growth in the brain environment, should be preferred over simpler models. These studies also include survival endpoints alongside delivery concentration data to confirm therapeutic efficacy. Research indicates that high drug delivery rates can fail to translate into clinical success if the agent lacks the potency to improve survival in models.

### 4.3. Tumor and Margin Pharmacokinetics

Another important factor is the evaluation of tumor and tumor margin pharmacokinetics. As we mentioned before, many studies report drug distribution in the whole brain. This, however, is insufficient for assessing therapeutic relevance. Effective translation must show adequate drug concentrations inside of the tumor and its infiltrative margins, where residual disease often stays. Preclinical studies should also include clinically meaningful comparisons. These may be systemic administration of the same drug, intranasal delivery of free drug, or appropriate device controls. Without these comparisons, it is difficult to determine whether a given system offers a true advantage over existing approaches.

### 4.4. Manufacturability Indicators

Finally, early indicators of manufacturability are equally important. Studies should report batch reproducibility and stability under storage and administration. These factors are all essential for scaling and regulatory approval, and their lack can greatly hinder a drug’s translation.

## 5. Tier Classification of Nanomaterial Platforms

To advance intranasal nanomaterial-based therapies for GBM, it is crucial that platforms which demonstrate realistic FIH potential must be identified [178,179]. Despite several nanocarrier systems demonstrating promising efficiency, only a small portion of these systems can effectively balance an approach that considers manufacturability, regulatory feasibility, safety, and clinical practicality [179]. Thus, these nanomaterial platforms can be categorized into three tiers which reflect their near-term clinical readiness and address translational obstacles including residence time (H2), tumor pharmacokinetics (H5), safety (H6), and quality control (H7). These sit alongside anatomical deposition (H1), mucus penetration (H3), dose and nasal-volume constraints (H4), and regulatory pathway and comparative benefit complexity (H8), which together make up the complete H1–H8 translational hurdle framework shown in Figure 1 (Section 2) and referenced throughout this review.

Tier assignment methodology: tier placement for each platform reflects two independent axes, each scored qualitatively as High, Moderate, or Low. The first axis, FIH readiness, reflects existing manufacturing, regulatory, or clinical precedent (e.g., FDA-approved analogs using the same platform class) and demonstrated formulation stability under storage and administration. The second axis, hurdles addressed, reflects how many of the eight translational hurdles defined in Section 2 (H1–H8) a platform mitigates without introducing new ones. A platform’s tier follows directly from how many of these two axes it scores High on: Tier 1 platforms score High on both axes, Tier 2 platforms score High on one axis, and Tier 3 platforms score High on neither. Table 1 (Section 3) summarizes FIH readiness, tier, and the specific hurdles (H1–H8) each platform addresses or leaves unresolved.

### 5.1. Tier 1: Most Realistic Candidates for Near-Term Translation

**Figure 3:**
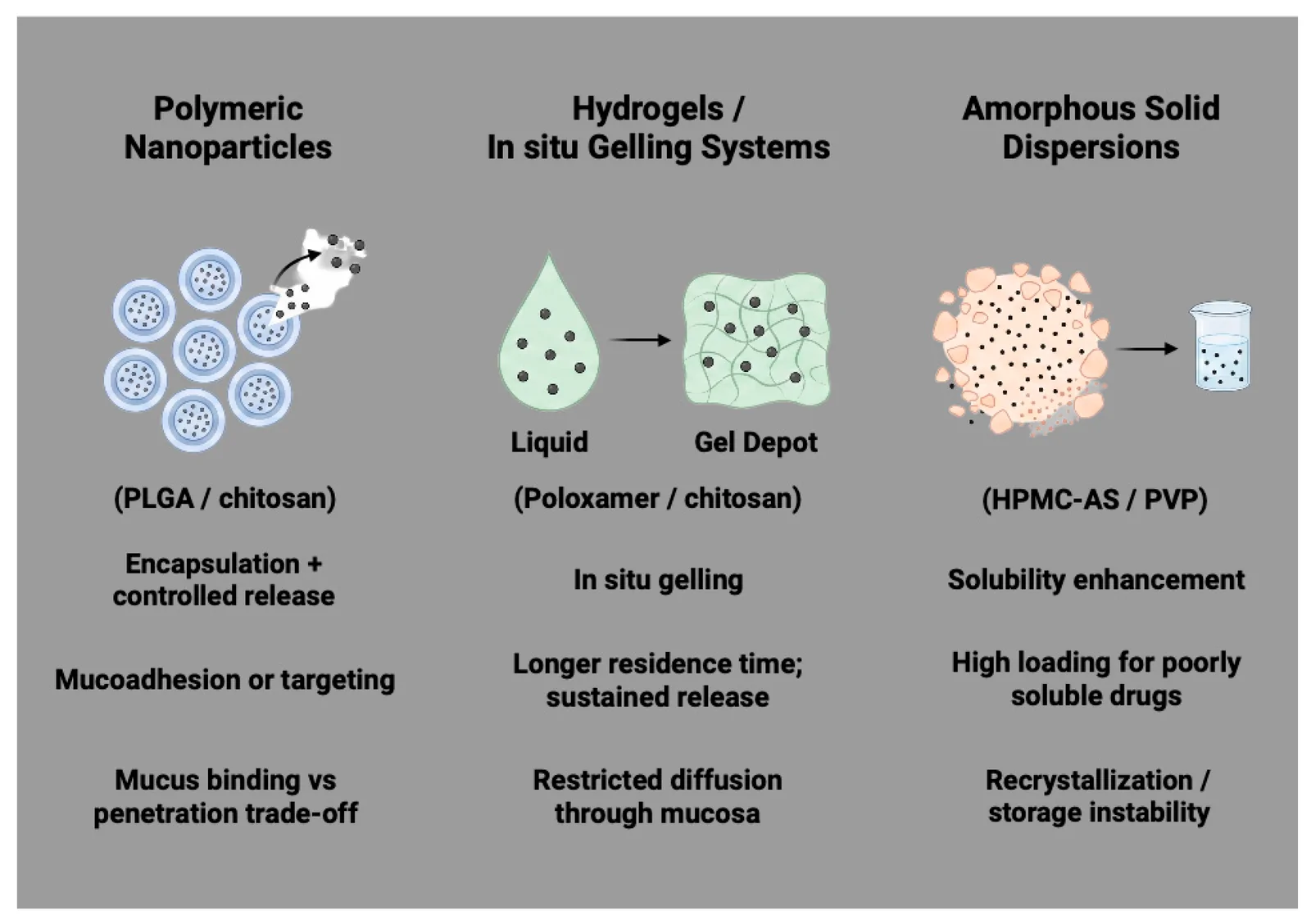
Tier 1 first-in-human readiness intranasal delivery systems. Depicted are Tier 1 FIH intranasal delivery systems including polymeric nanoparticles, hydrogels and amorphous solid dispersions. Created in BioRender. Keniry, M. (2026) https://BioRender.com/ltixy7z.

In Tier 1, nanomaterial systems are characterized as the most realistic candidates for near-term clinical translation [139,180]. This is due to their established safety protocols and manufacturing simplicity. One system that falls into this category is simple, biodegradable polymeric nanoparticles (BPNPs), which provide a strong balance between flexibility in drug loading and manageable safety, manufacturing, and controls [181,182] (see Table 1, Polymeric nanoparticles). Additionally, hydrogels and in situ gelling nanoparticles fall into this tier as they offer a direct solution to the barrier of limited nasal residence time (H2) (see Table 1, Hydrogels). Furthermore, amorphous solid dispersion (ASD)-based powders have also demonstrated strong translational potential, especially in the case of poorly soluble drugs, since they can enable high dose efficiency within the limited intranasal volume while simultaneously maintaining shelf stability [165,166] (see Table 1, and Figure 3).

### 5.2. Tier 2: Realistic but Requiring Additional Development

In Tier 2, nanomaterial systems are considered realistic but require additional development to address translational barriers and move from bench to clinical settings [110,125]. For example, polymeric micelles—specifically those with simplified architectures, such as diblock (AB) or triblock (ABA) copolymers—are well-suited for hydrophobic drug delivery but must still meet stability standards related to dilution within the bloodstream and addressing physical stress in aerosolization conditions [109,122] (see Table 1, Polymeric micelles).

LNP sprays and LNP-loaded mucoadhesive fibers are also included within this tier due to their advantages in payload versatility and manufacturing control; however, their effectiveness depends on efficient nasal tolerability as well as their ability to navigate regulatory pathways [25]. Studies have shown that residence time can be improved by including mucoadhesive fibers and/or coatings to LNPs [26,27]. However, challenges consist of side effects, damaging cilia, and generating contact time that can overcome the mucociliary clearance mechanism [26,27] (see Table 1, Lipid nanoparticles).

Furthermore, mucoadhesive fibers (or inserts), especially those which utilize micelles or nanoparticles, provide the advantage of improved deposition control and increased residence time [183]. These fibers use polymers which interact with mucin, which are glycosylated proteins that are responsible for mucosal structure [26,27]. By interacting with mucin, these fibers can reduce dose frequency by increasing drug contact time, ultimately enhancing drug bioavailability through adhesion to the mucosal layer [26,27]. These systems, overall, are promising but require additional comprehensive developmental adjustments to support FIH studies. To be considered for testing in FIH studies, these mucoadhesive fibers currently lack standardized methodology and long-term studies that focus on stability and toxicity, which are needed for clinical translation [95,184].

### 5.3. Tier 3: Least Realistic for Near-Term Intranasal Oncology Trials

In Tier 3, these nanoparticle systems are considered the least realistic for near-term intranasal oncology trials due to their significant safety and translational challenges. AuNPs fall into this category due to their inorganic nature, which raises concerns of long-term accumulation and chronic toxicity (H6), as well as clearance and combination-product regulatory burden (H8). These concerns increase the burden and risk of not obtaining regulatory approval (see Table 1, Gold nanoparticles).

Similarly, protein-based nanocages (such as ferritin) offer the advantage of strong biological targeting potential yet are severely limited due to their issues related to structural heterogeneity, complex manufacturing, and immunogenicity [185]. Protein-based nanocage methodology includes expression systems, such as *E. coli* and mammalian cells, but requires tedious optimization leading to constant extensions of development timelines [62,186]. These characteristics significantly affect the reproducibility of protein-based nanocages and add to complications in quality control (H7) [185]. Overall, these systems are generally unlikely to advance into early-phase clinical trials without first addressing safety and manufacturability concerns (see Table 1, Ferritin nanocages).

### 5.4. Summary

As a result, nanoparticle systems which prioritize reproducibility and execute simple manufacturing processes are more likely to meet safety standards and be suitable for early clinical trials. Advancing research within this field heavily depends on addressing translational challenges and aligning nanomaterial designs with regulatory expectations, which would progress these systems towards achieving therapeutic tumor exposure.

## 6. Discussion

Glioblastoma is a devastating cancer in which patients survive fourteen months on average [1]. This cancer is infiltrative with strong migration capabilities and has heterogeneous cell types within a given tumor, making efficacious treatment difficult [187]. Glioblastoma harbors an embryonic stem cell signature, in part making it resistant to conventional chemotherapeutics [188]. This review discusses the possibility of intranasal delivery systems as a mechanism to get therapeutics to brain tumors. There are many different techniques in this emerging field that have been discussed in this review detailing the respective strengths and weaknesses of each application, such as drug solubility, stability, targeting, or bioavailability. Promising methodologies that would likely be the first-in-human applications include polymeric nanomaterials, hydrogels, and ASDs. Despite encouraging preclinical outcomes, clinical translation remains limited due to challenges including variability in nasal anatomy and physicology, mucociliary clearance, limited dose capacity, formulation stability, and scalability, as well as a lack of ability to accurately predict human nose-to-brain transport. There are issues with manufacturing reproducibility that make the scalability of this application problematic. The amount of drug loaded into these delivery systems and the structure of the carriers themselves need to be reproducible as many chemotherapeutics can be toxic, even lethal at elevated doses, making the need for careful manufacturing imperative. Furthermore, testing the delivery methods must move away from rats and mice, which appear to have a greater ability to absorb therapeutics and give an inflated picture of how well intranasal delivery works [75]. Some studies are using pigs as a model system to investigate intranasal delivery methods [189]. There are promising interventions under development that may make intranasal delivery methods more precise, such as advanced intranasal delivery devices, breath-powered systems or precision nasal applicators [68,69].

### Extension Beyond Glioblastoma

Although this review focuses primarily on GBM, the therapeutic principle of delivering nanomaterials through the nose in order to bypass the BBB via direct nose-to-brain pathways can be applied to many other clinical conditions. For example, other primary CNS cancers, such as medulloblastoma, meningioma, and diffuse midline glioma, face the same main obstacle of poor drug penetration through the BBB. These conditions could very well profit from these delivery platforms; however, concrete laboratory and clinical evidence for these conditions is currently very sparse and requires further studies. A particularly urgent and rapidly growing area of application focuses on metastatic brain tumors that originate from primary lung, breast, melanoma, or colon cancers [190]. These secondary malignancies are significantly more common than primary brain tumors and face physicological delivery barriers that intranasal nanomedicines are specifically engineered to bypass. Outside of oncology, researchers are exploring nose-to-brain administration for long-term brain disorders and show promising results in Alzheimer’s and Parkinson’s disease [107,191].

However, these delivery platforms vary in how they adapt to different diseases. Hydrogel and ASD systems are considered to be generally versatile across conditions due to their extended retention time and enhanced deposition within the nasal cavity. Consequently, adapting these systems for a new brain condition requires substituting the active pharmaceutical ingredient with minimal structural modifications. However, on the other hand, systems which use active molecular targeting, such as ferritin nanocages, rely heavily on binding to specific cell receptors. Since the surface markers on glioblastoma cells differ fundamentally from metastatic cancer cells or damaged neurons found in Alzheimer’s disease, these targeted systems require customized redesigns of their targeting mechanisms for every distinct tumor type or disease.

These extensions are not equally supported by the H1-H8 framework developed earlier within this review. Brain metastases and primary CNS malignancies share H1 (anatomical deposition) and H5 (tumor-specific pharmacokinetics) with glioblastoma, since both conditions require transport to the olfactory region and achieving adequate concentration. Pediatric conditions such as medulloblastoma and diffuse midline glioma introduce an additional translation gap beyond H4 (dose constraint), which is that pediatric nasal anatomy differs from adult nasal anatomy underlying most of the deposition and residence time within this review, meaning that dosing volume cannot be easily adjusted from adult studies. Furthermore, non-oncological CNS conditions such as Alzheimer’s and Parkinson’s have different targets, such as amyloid-beta plaque, rather than a tumor cell surface receptor. Thus, platform-level generalizability and disease-level generalizability are separate claims in the sense that a platform can be broadly useful, whereas a specific targeted formulation cannot.

*Diffuse midline glioma:* known to be highly aggressive, resistant, and unresectable due to its originating in the brainstem, diffuse midline glioma treatment is significantly limited by the BBB. Due to its anatomical position being within proximity of the trigeminal nerve, developing a nose-to-brain pathway is attractive for targeting an inoperable tumor; however, it also introduces the hurdle of adult-to-pediatric scaling. Current research is investigating the use of nanoparticles in diffuse midline glioma through cell-penetrating peptides, magnetic drug delivery, carbon dots, neoantigens and tumor-associated antigens, and tumor targeting bacteria [192]. Preclinical studies show promising results, with enhanced uptake and anti-tumor effects with cell-penetrating peptides, mutation-specific and antigens demonstrating safety and efficacy signals, and tumor suppression and improved survival with tumor targeting bacteria [192]. Multiple clinical trials are ongoing, with quality of life and minimizing toxicity being of utmost priority in developing new therapies. However, direct evidence of intranasal nanomaterial delivery for diffuse midline glioma remains absent from the literature, and focus more on cell-based carriers.

*Medulloblastoma:* as the most common malignant pediatric brain tumor, medulloblastoma treatment is complicated by the BBB, severely limiting the effectiveness and bioavailability of many drugs. As a result of this poor bioavailability, higher systemic doses may be needed, which would result in an increase in toxicity [193]. Current research on nanomaterial applications in medulloblastoma is primarily focused on intravenous administration as opposed to intranasal delivery, utilizing a fluoropolymer-engineered magnetic iron-oxide nanoparticle carrying siRNA [193]. Results demonstrated the ability to cross the BBB and, once delivered, to effectively silence oncogenic gene expression in medulloblastoma models [193]. These results confirm that using nanoparticles to target medulloblastoma is not only practical, but biologically achievable. However, translating an intravenous application to an intranasal application requires reformulation and evaluation of safety and toxicity assessments.

*Meningioma:* unlike GBM, meningiomas are extra-axial tumors which originate from the arachnoid cap cells of the meninges, as opposed to the brain parenchyma, as seen in GBM. Meningiomas are characterized by vasculature that is intrinsically leaky and is not protected by the BBB [194]. Due to this characteristic, meningiomas demonstrate avid and homogenous contrast enhancement upon imaging, as opposed to GBM, which typically demonstrate a patchy enhancement pattern [194]. Thus, the main argument of this review, that nose-to-brain delivery is advantageous as it bypasses the BBB, is not as applicable or relevant for meningioma due to their naturally leaky vasculature and lack of a protective BBB, which makes the need to circumvent the BBB less critical for drug delivery. Meningiomas are commonly treated with surgery or radiation for the reason that systemically administered drugs are able to access these tumors effectively [194]. Therefore, the main clinical challenge is not bypassing the BBB, but rather minimizing the systemic drug exposure that occurs in the minority of atypical or malignant meningiomas that need pharmacological therapy.

*Brain metastases*: many primary cancers metastasize to the brain, with over 50% of lung cancers, 15–25% of breast cancers, and 5–20% of melanoma, kidney, colorectal, testicular and thyroid cancers also contributing to metastatic brain cancer. Patients with a primary cancer have a 20–40% chance they will develop brain metastases and usually have an overall survival rate of 1–3 months. Brain metastases are difficult to treat since they depend on multiple factors, including type of primary cancer, how the patient tolerated radiotherapy and chemotherapy, clinical history, and importantly, how many metastases there are [195].

*Alzheimer’s disease:* recent Alzheimer’s disease research has been investigating nanoparticle delivery systems such as polymeric nanoparticles, lipid-based carriers, and intranasal nano formulations, with modifications to reduce neuroinflammation and improve mitochondrial function to rescue the energetic crisis that Alzheimer’s causes. Challenges for these therapeutics are much the same as the challenges for GBM with scalability, safety validation, and clinical translation [196].

*Parkinson’s disease*: experimental and preclinical studies show neurotrophic factors support neuroprotection against oxidative stress, mitochondrial dysfunction, and neuroinflammation. During preclinical trials, proper safety levels and expected results were achieved, but during early-phase clinical trials, the studies struggled with the efficacy of the treatment due to constraints of brain delivery, invasive procedures, and patient heterogeneity [197]. The overarching theme of needing a safe, effective and scalable delivery platform for any of these detrimental diseases dealing with the blood–brain barrier shows great promise for any therapeutic that can achieve these requirements.

## 7. Conclusions

Nose-to-brain chemotherapy will be clinically successful when it is engineered as a three-part structure that considers (1) route, (2) dosage form, and (3) device system (likely requiring an advanced delivery system). The system would have to clear the dominant hurdle for its respective drug since we have pointed out which techniques are suitable for stability, deposition, residence, and scalability. The system must also provide tumor-relevant pharmacokinetics and survival benefit compared to the current standard of care. Intranasal delivery methods offer much potential for delivering therapeutics to the central nervous system. While the focus of this review was primarily on the treatment of glioblastoma, this research and its applications also would have a great effect on the treatment of CNS disorders and neurodegenerative diseases that require efficient treatment to pass the blood–brain barrier. Likewise, these described delivery mechanisms could also be applied to a host of other cancers that target the brain, including metastases of breast, colon, melanoma, and lung cancer [190]. This review has highlighted the key biological and engineering challenges that must be addressed in the design and development of a more efficient drug delivery system, and as discussed above, the same challenges and platform logic extend beyond GBM to other CNS malignancies, brain metastases, and neurodegenerative and neurological disease. A translation focused approach that can integrate efficacy, safety, and scalability offers the most credible path to successful clinical trials for intranasal nose-to-brain chemotherapy.

## Data Availability

No new data were created or analyzed in this study. Data sharing is not applicable to this article.

## Abbreviations

The following abbreviations are used in this manuscript:

AB	Diblock copolymer (A–B architecture)
ABA	Triblock copolymer (A–B–A architecture)
AI	Artificial intelligence
APS	Amorphous phase separation
ASD	Amorphous solid dispersion
AuNP	Gold nanoparticle
BBB	Blood–brain barrier
BCS	Biopharmaceutics classification system
BPNP	Biodegradable polymeric nanoparticle
CMC	Critical micelle concentration
CNS	Central nervous system
CS	Chitosan
DOX	Doxorubicin
EDY	Maleimidenediyne
FDA	U.S. Food and Drug Administration
FIH	First-in-human
FNP	Functionalized nanoparticle
GBM	Glioblastoma
GEM	Gemcitabine
H1–H8	Translational hurdles 1–8
HPMC	Hydroxypropyl methylcellulose
HPMCAS	Hydroxypropyl methylcellulose acetate succinate
LNP	Lipid nanoparticle
MRI	Magnetic resonance imaging
MTD	Maximum tolerated dose
NLC	Nanostructured lipid carrier
NP	Nanoparticle
PCL	Polycaprolactone
PD-L1	Programmed death-ligand 1
PEG	Polyethylene glycol
PET	Positron emission tomography
PK	Pharmacokinetics
PLA	Polylactic acid
PLGA	Poly(lactic-co-glycolic acid)
PM	Polymeric micelle
PMPC	Poly(2-methacryloyloxyethyl phosphorylcholine)
PVA	Polyvinyl alcohol
PVDF	Polyvinylidene fluoride
PVP	Polyvinylpyrrolidone
RNAi	RNA interference
SCARA5	Scavenger receptor class A member 5
SLN	Solid lipid nanoparticle
SPECT	Single photon emission computed tomography
TfR1	Transferrin receptor 1
Tg	Glass transition temperature
TIM-2	T-cell immunoglobulin and mucin domain 2
